# On Arsenic in Cutaneous Diseases

**Published:** 1858-09

**Authors:** John R. Cushing

**Affiliations:** South Butler, Ala.


A T L A N T A

### Medical and Surgical Journal.

Vol. IV.] SEPTEMBER, 1858. [No. I.

### ORIGINAL COMMUNICATIONS.

### ARTICLE I.

*On Arsenic in Cutaneous Diseases.* By John R. Cushing, M. D., South Butler, Ala.

I forward to the "*Journal,*" a few cases strongly illustrative of the value of Arsenic, in inveterate skin diseases.

The Profession has been so much carried away by the use of the Iodide of Potassium, Sarsaparilla, and Taraxacum, during the last twelve or fifteen years, that this invaluable and good old remedy has been greatly neglected.

No one will more readily acknowledge than I do, the great remedial power of the above standard remedies, and I fully concede, that we have no better alterative than the Iodide in many chronic diseases, but I do not believe in running a good thing in the ground, and making it *sine dubio* the great specific for everything.

Yet, you, I, and all of us, have frequently met with mortifying disappointments in its use, where we have anticipated entire success, especially in old and obstinate cutaneous diseases, where it fails in many instances. This paper, therefore, is not written to propagate an original "idea" to the Medical World, but merely to revive a long neglected and invaluable Therapeutical agent, for I deem it to be the duty of every Practitioner, when he has cured, or any way ameliorated a helpless victim of a supposed incurable disease, to bring it before his professional brethren.

Arsenic, we all know, is an old, and thought to be hazardous remedy for many diseases, and owing to its *terribly* poisonous properties has been but little used by the profession generally, and since the introduction of Quinine and the Iodide, it has been almost wholly neglected, though never popular at any time. But to the cases.

*Prurigo.*

Case 1.—Mr. R——, aged forty-nine, of a highly nervous temperament, consulted me for an obstinate pruritus round the verge of the anus, perineum, scrotum, thighs, etc. He stated he had tried a "thousand remedies," from which he only derived temporary relief, and latterly had been taking opiates, and hydrocyanic lotion to relieve the intolerable itching, the Iodide had been used to no effect, the parts were excoriated and inflamed.

He was treated with the solution of arseniate of potassa gtt v three times a day in Lager Beer. Cured in forty-one days.

Case 2. Mrs. J——, aged thirty-two, applied to me for violent Pruritus of the genital parts, had used many remedies to no effect, itching intolerable, excoriation and inflammation of the parts, no enjoyment to self, family or friends. Treatment, Fowler's solution as above, only commencing with three drops and increasing every week up to *five. Cured in nine weeks.*

These, with many other cases I could cite, of skin diseases, especially Sycosis, Acne, Rosacea, Rupia, etc., have met with prompt success and perfect cure.

Not only in cutaneous diseases are the arsenical preparations valuable, but I know of no better remedy than the following in obstinate Intermittents, I have used it often, when quinine and everything else failed, and therefore recommend it as infallible.

| | | |
|---|---|---|
| ℞ | Sulph. Quinine ........ .................... | ʒij. |
| | Fowler's Solution............................ | ℥ss. |
| | P. P. Ferri.................................... | ℥ss. |
| | Camph. Water.............................. | ℥viii. |

Tea spoonful three times a day in half tumbler water. I sometimes substitute Donovan's solution, when a mercurial alterative is wished.

In the use of arsenic, I would state, that there is always a stopping point, especially in long standing cases, where the preparation has to be used for an indefinite period, and in the observance of that point, no danger need ever be apprehended of its poisonous influence, and that point is the peculiar puffiness it occasions in the eyelids. When this is observed, desist immediately until it disappears, then resume again.

